# Isolated Fallopian Tube Torsion with Necrotic Hemorrhagic Cyst in an 11-Year-Old Girl Diagnosed by Laparoscopy

**DOI:** 10.1155/2023/8928662

**Published:** 2023-08-09

**Authors:** Atl Simon Arias Rivera, Luis Miguel Zamora Duarte, Samuel Shuchleib Chaba, Alberto Chousleb Kalach, Marcos Jalak Cababie, Ariel Shuchleib Cung

**Affiliations:** Department of General Surgery, Hospital Angeles Lomas, Mexico City, Mexico

## Abstract

Isolated fallopian tube torsion (IFTT) is a rare pathology that causes acute abdomen in women, it is even less common in pediatric patients. We present a case of an 11-year-old girl who presented with abdominal pain 24 hours of evolution, the diagnosis could not be specified with cabinet methods, so the definitive diagnosis was made using a diagnostic laparoscopy. A necrotic hemorrhagic tubal cyst was found. A left salpingectomy had to be performed due to necrosis. Early diagnosis can have a positive impact on the fertility of these patients.

## 1. Introduction

Isolated fallopian tube torsion (IFTT) is a rare cause of gynecological acute abdomen and was first reported by Bland-Sutton. The estimated prevalence is 1 in 1.5 million women [1]. Unfortunately, the diagnosis can be difficult because of aspecific symptoms. The delay in the management of this pathology can have important implications since the torsion of the tubes can cause ischemia and necrosis of these [2]. IFTT occurs mainly in women of reproductive age, it is rare in pediatric populations or postmenopausal women [3]. The clinical findings of tubal torsion are varied and nonspecific and include severe, sharp lower abdominal pain, often accompanied by nausea and vomiting, but without specific symptoms. We present the case of a patient in early puberty who presented with acute abdominal pain due to a complete torsion of the left fallopian tube, which resulted in necrosis of the left fallopian tube secondary to a hemorrhagic cyst [4].

## 2. Case Presentation

An 11-year-old female with no significant medical history came to the emergency room with severe abdominal pain with an evolution of 24 hours, the pain was found to be accompanied by nausea and vomiting.

Physical examination revealed pain on palpation in the hypogastrium, which radiated towards the right iliac fossa, even though in the final diagnosis, the pathology was left accompanied by signs of localized peritoneal inflammation. Within the laboratory studies, she presented a leukocytosis of 14,400 at the expense of neutrophilia. The transabdominal ultrasound showed decreased peristalsis, with positive sonographic McBurney. Presence of abundant free fluid towards the right iliac fossa and pelvic cavity.

**Figure 1:**
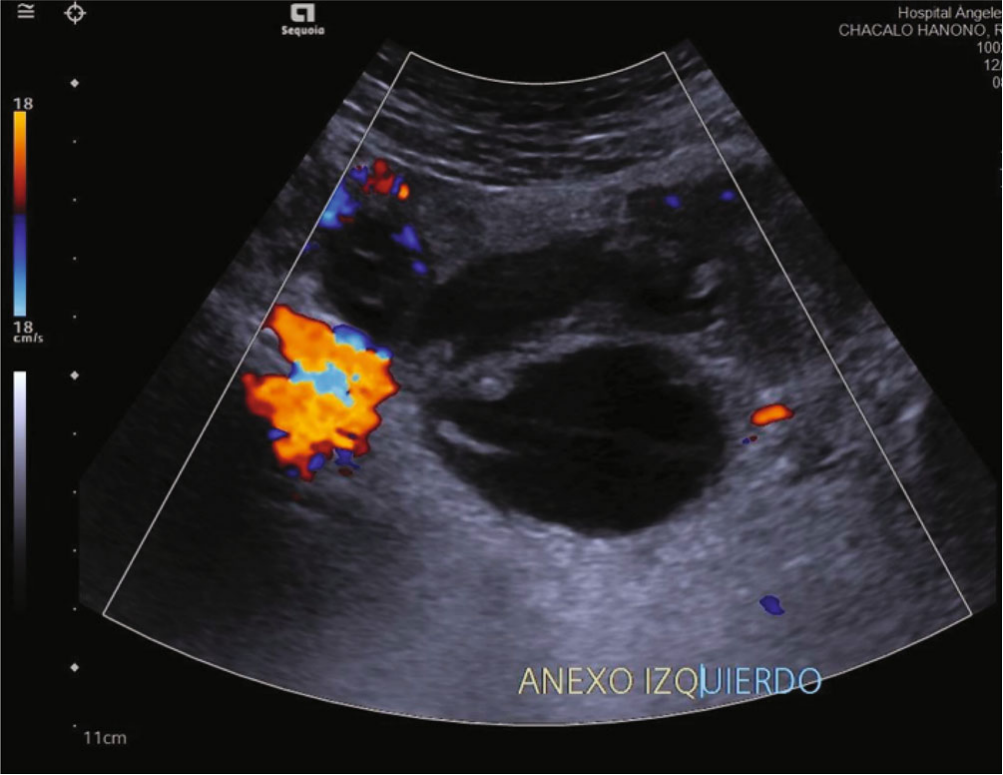
Collection in the pelvic cavity of heterogeneous content, without vascularity on color Doppler of 5.9 cm × 5.6 cm × 4.9 cm with an approximate volume of 86 cc.

**Figure 2:**
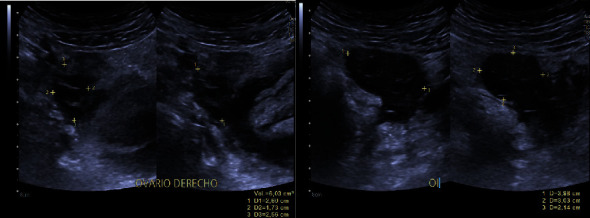
The right ovary had a calculated volume of 6 ml and the left 12.6 ml, both with normal characteristics and no evidence of focal lesions.

A 5.9 cm × 7.2 cm × 4.9 cm collection in the pelvic cavity is evidenced, with heterogeneous content, without vascularity after the application of color Doppler, with an approximate volume of 86 cc (Figure 1). The right ovary had a calculated volume of 6 ml and the left 12.6 ml, both with normal characteristics and no evidence of focal lesions (Figure 2).

**Figure 3:**
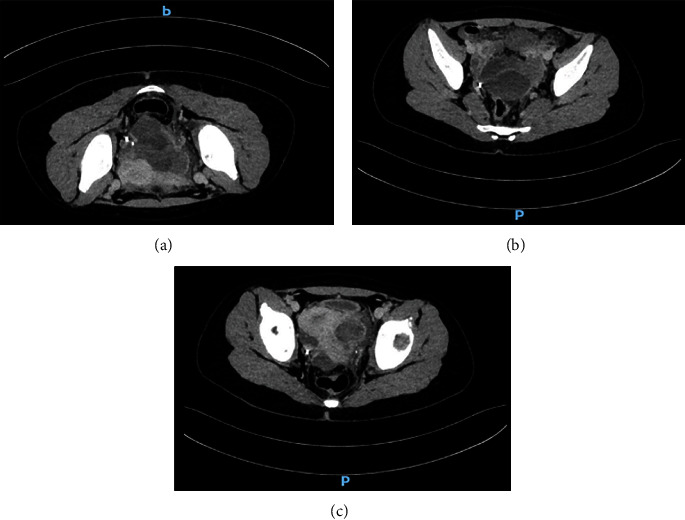
(a–c) Multilocular collection, with heterogeneous liquid content that peripherally enhances with contrast medium, measures 5.6 cm × 7.2 cm × 5.5 cm.

Due to the lack of an accurate diagnosis for this collection, an abdominal tomography with intravenous contrast was performed, which showed a multilocular collection, with heterogeneous liquid content (U.H. 16–22), with dimensions similar to those previously described, which, was found in contact with the ovaries but did not appear to originate from them, free fluid in the pelvic cavity and striation of the intraperitoneal fatty tissue of the pelvic level (Figure 3).

At that time, some of the differential diagnoses considered were tubo-ovarian abscess, complicated chemical rupture, and pelvic abscess due to some non-gynecological causes, such as perforated Meckel's diverticulum, among others.

**Figure 4:**
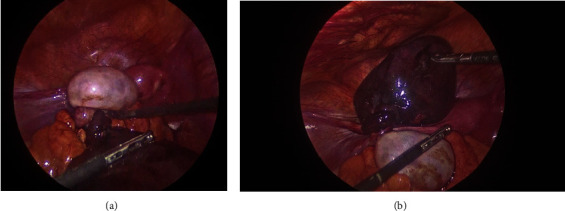
(a and b) These images show the laparoscopic view, in which the presence of the enlarged ovary can be seen, as well as the presence of the twisted fallopian tube, from which a necrotic/hemorrhagic cyst emerges.

Diagnostic laparoscopy was performed to determine the origin of the pelvic collection and the symptoms, in which free fluid was identified in the pelvic cavity with serohematic characteristics, as well as a necrotic tubal cyst with the vascular compromise of the fallopian tube (Figure 4). A left ovarian cyst with a follicular appearance was also discovered, apparently in a retrocecal position, without alterations.

Torsion of the left fallopian tube was reversed; however, given that it was found to be necrotic a resection was performed, preserving both ovaries.

The pathology report confirms the diagnosis of the fallopian tube with accentuated hemorrhagic necrosis of the wall, in addition to vascular congestion with blood clots and formed blood elements.

The patient had an adequate post-surgical evolution and recovered without alterations, especially in the future it may have repercussions on fertility, however, since he has both ovaries and an intact tube, it is expected that it will not have major repercussions.

## 3. Discussion

IFTT is a rare cause of abdominal or pelvic pain in young women, the differential diagnosis in the pediatric and adolescent population includes acute appendicitis, pelvic inflammatory disease, twisted ovarian cyst, ruptured follicular cyst, urinary tract disease, and renal colic [5]. This circumstance is extremely rare before menarche [6]. It occurs more commonly on the right side (around 60%), unlike our case, which was on the left side [7].

Mazouni et al. [8] showed that the risk of adnexal necrosis increases significantly when the delay time for surgery is greater than 10 hours. An earlier study also found that patients with pain for more than 24 hours were more likely to undergo salpingectomy, suggesting that longer periods of torsion may lead to greater tissue necrosis [2].

The etiology of IFTT is still uncertain, especially when it is not associated with ovarian torsion. Suggested mechanisms include anatomic malformations, such as long mesosalpinx, Morgagni's hydatids, and hydrosalpinx; abnormalities in adjacent organs, such as ovarian and paraovarian masses, uterine enlargement due to pregnancy or tumor, and peritubal adhesions; physiological alterations in the normal peristalsis of the tube; hemodynamic alterations that cause congestion and tortuosity of the mesosalpingeal veins; sudden changes in body position leading to abnormal adnexal movement; accident trauma; and previous tubal operations, such as sterilization [4].

Risk factors for IFTT include intrinsic factors, including pelvic inflammatory disease, hydrosalpinx, tubal ligation, and tubal neoplasia; and extrinsic factors, such as adhesions, adnexal venous congestion, adjacent ovarian or paraovarian masses, uterine masses, gravid uterus, and trauma [9].

Our patient did not have any of the above risk factors for torsion at presentation, however, she did have a large tubal cyst, which likely facilitated torsion.

The first diagnostic imaging that has to be performed on a pubertal girl with abdominal pain should be the ultrasound. The presence of an adjacent normal ovary, separate from a cystic structure could assist in preoperative suspicion of IFTT [10].

Treatment options include surgical detorsion, salpingostomy, and salpingectomy depending on the stage and presence of operative complications.

Diagnostic laparoscopy is essential in these patients because it allows rapid diagnosis and early treatment with low morbidity. It is important that, if resources and training are available, the intervention is performed laparoscopically, since this reduces adhesions in the pelvis when compared with open surgery. This can have a positive impact on fertility, especially since this pathology occurs mainly in women of childbearing age [7].

## 4. Conclusion

The IFTT is a rare surgical emergency, however, it should be considered in the differential diagnosis of adolescent women with severe pelvic pain in women of childbearing age, since the delay in diagnosis and management of this pathology can have significant repercussions on the patient's fertility.

The earlier the intervention, the greater the probability of being able to rescue the affected tube.

## Data Availability

All the data is available on the http://pubmed.com website with the references cited in the text.
